# MicroRNA-146 function in the innate immune transcriptome response of zebrafish embryos to *Salmonella typhimurium* infection

**DOI:** 10.1186/1471-2164-14-696

**Published:** 2013-10-10

**Authors:** Anita Ordas, Zakia Kanwal, Valesca Lindenberg, Julien Rougeot, Matyas Mink, Herman P Spaink, Annemarie H Meijer

**Affiliations:** 1 Institute of Biology, Leiden University, Einsteinweg 55, 2333 CC Leiden, The Netherlands; 2 Department of Genetics, Faculty of Science and Informatics, University of Szeged, 52 Kozepfasor, H-6726 Szeged, Hungary

**Keywords:** MicroRNA, miR-146, Innate immunity, Infection, *Salmonella typhimurium*, *Mycobacterium marinum*, MyD88, Traf6, Apolipoproteins, Zebrafish

## Abstract

**Background:**

MicroRNAs (miRNAs) have recently been shown to play important roles in development of the immune system and in fine-tuning of immune responses. Human miR-146 family members are known as inflammation-inducible miRNAs involved in negative feedback regulation of Toll-like receptor (TLR) signalling. Dysregulation of the miR-146 family has often been linked to inflammatory diseases and malignancies. This study reports on miR-146a and miR-146b as infection-inducible miRNAs in zebrafish, which has emerged as a model species for human disease.

**Results:**

Using a custom-designed microarray platform for miRNA expression we found that both members of the zebrafish miR-146 family, miR-146a and miR-146b, were commonly induced by infection of zebrafish embryos with *Salmonella typhimurium* and by infection of adult fish with *Mycobacterium marinum*. The induction of these miRNAs was confirmed by Taqman miRNA assays. Subsequently, we used zebrafish embryos, in which adaptive immunity is not yet active, as an *in vivo* system to investigate the role of miR-146 in the innate immune response to *S. typhimurium* infection. Knockdown of *traf6* and use of *myd88* mutants demonstrated that the induction of miR-146a and miR-146b by *S. typhimurium* infection was affected by disruption of the MyD88-Traf6 pathway that mediates transduction of TLR signals and cytokine responses. In turn, knockdown of miR-146 itself had no major effects on the expression of known targets of MyD88-Traf6 signalling. Instead, RNA sequencing analysis showed that miR-146 knockdown led to an increased induction of six members of the apolipoprotein gene family in *S. typhimurium*-infected embryos.

**Conclusion:**

Based on microarray analysis and Taqman miRNA assays we conclude that members of the miR-146 family, which is highly conserved between fish and human, are induced by bacterial infection in zebrafish in a MyD88 and Traf6 dependent manner. The combined knockdown of miR-146a and miR-146b in zebrafish embryos infected with *S. typhimurium* had no major effect on the expression of pro-inflammatory genes and transcription factors known to be downstream of the MyD88-Traf6 pathway. In contrast, apolipoprotein-mediated lipid transport emerged as an infection-inducible pathway under miR-146 knockdown conditions, suggesting a possible function of miR-146 in regulating lipid metabolism during inflammation.

## Background

Timely activation as well as termination of inflammatory responses is essential for proper functioning of the immune system. A balanced output of the vertebrate immune response is dependent on several regulatory mechanisms, in which microRNAs (miRNAs) have recently emerged as new players with critical importance [1]. Several human diseases, including cancer, autoimmune diseases, and chronic inflammatory disorders, have been associated with dysregulation of miRNA expression both in a positive or negative regulatory manner [1-5]. MiRNAs are evolutionary conserved, genome-encoded small RNAs (~22 nucleotides) that down-regulate gene expression at the post-transcriptional level by either translational repression or by mRNA degradation through binding to the 3′-UTR of their target mRNAs [6]. MiRNAs were found to have roles in a diverse range of processes ranging from development, cellular differentiation, hematopoiesis, apoptosis, and growth, to the functioning of the immune system [7-10].

The role of host miRNAs in bacterial infections has been addressed in several previous studies. MiRNAs were observed to be differentially regulated by Toll-like receptor (TLR)-mediated recognition of bacterial molecules. For instance, lipopolysaccharide (LPS) stimulation of TLR4 and downstream NFκB activity induced miR-146, miR-147, and miR-155 [11-13]. Mice deficient in miR-155 could not be protected by vaccination against *Salmonella typhimurium* infection and showed strong defects in T-cell cytokine production [14]. In another study, *Salmonella typhimurium* infection was found to induce rapid down-regulation of let-7 miRNA family members in macrophages, thereby leading to an upregulation of let-7 targets, the IL-6 and IL-10 cytokines [15]. IL-10 production in LPS-stimulated macrophages was also found to be regulated by miR-98 [16]. Furthermore, miR-29 was found to suppress immune responses to *Listeria monocytogenes* and *Mycobacterium tuberculosis* by targeting IFN-γ [17].

The LPS-inducible miR-146 family comprises two members in human, miR-146a and miR-146b, which are transcribed from different genes on chromosome 5 and 10, respectively [18]. They have the same seed sequence (i.e. the sequence essential for the binding of the miRNA to its mRNA target) and differ in their mature sequence by only two nucleotides at the 3′end. Dysregulation of miR-146a and miR-146b has been observed in many types of malignant tumors and several studies implicate these miRNAs as metastasis suppressors [18-23]. MiR-146a has also been implicated in different autoimmune diseases, including systemic lupus erythematosus, rheumatoid arthritis and Sjögren’s syndrome [24,25]. The first indication of the role of miR-146a/b in innate immunity came from work of Taganov et al. [11], showing increased expression of these miRNAs in the human monocytic THP-1 cell line when triggered by LPS. Promoter analysis revealed that induction of the miR-146a gene by LPS, TNFα, and IL-1 is mediated by the NFκB transcription factor [11]. In addition, 3′-UTR luciferase reporter assays demonstrated that the TLR signalling intermediates IRAK1 and TRAF6 are potential targets of miR-146a and miR-146b [11]. These data suggested that the miR-146 miRNAs function in a negative feedback pathway of TLR and cytokine signalling by targeting *IRAK1* and *TRAF6* mRNAs for down-regulation, a conclusion supported by recent analysis of miR-146a knockout mice [26]. MiR-146a has also been shown to function as a negative regulator of interferon (IFN) signalling by targeting the IRF5 and STAT-1 transcription factors [27] and to control the resolution of T-cell responses in mice [28].

The zebrafish provides a useful model to study innate immunity, which is the primary line of defence against infections during the first few weeks of development, when functional adaptive immunity is not yet present [29,30]. The zebrafish model combines an efficient genetic toolbox with excellent possibilities for high resolution imaging of host-microbe interactions at the early life stages (embryos and larvae), when zebrafish are transparent [29,30]. Many zebrafish infection models for bacterial pathogens have recently been developed, among which the *S. typhimurium* and *M. marinum* models are the best characterized [29]. The major signalling pathways of the innate immune system are conserved within all vertebrates [31] and the repertoire of zebrafish miRNAs is well described [32,33]. Since miRNAs are strongly conserved among all vertebrates, the advantages of the zebrafish model organism may be exploited to elucidate miRNA functions in the vertebrate host response to bacterial infections. As in mammals, the zebrafish miR-146 family has two members, named dre-miR-146a and dre-miR-146b, which are present within genes located on chromosome 13 and 21, respectively. The *IRAK1* and *TRAF6* homologs of both zebrafish and human contain putative target sites for miR-146 in their 3′-UTRs, suggesting that miR-146 feedback control of TLR signalling is evolutionary conserved. Here we report on a microarray study of miRNA expression, which showed that miR-146a and miR-146b are commonly induced by infection of zebrafish embryos with *Salmonella typhimurium* and by infection of adult fish with *Mycobacterium marinum*. We demonstrate the requirement of the MyD88-Traf6 pathway for the infection-triggered induction of miR-146a/b in the zebrafish embryo model. Furthermore, we used morpholino knockdown to suppress the function of miR-146a and miR-146b and analyzed the effects of this down-regulation by RNA deep sequencing (RNAseq) of embryos infected with *Salmonella typhimurium*. While no major effects on known targets of the MyD88-Traf6 pathway were observed, apolipoprotein-mediated lipid transport emerged as a novel infection-inducible pathway under control of miR-146a/b.

## Results

### Microarray analysis identifies infection-inducible miRNAs in zebrafish embryos and adults

**Figure 1:**
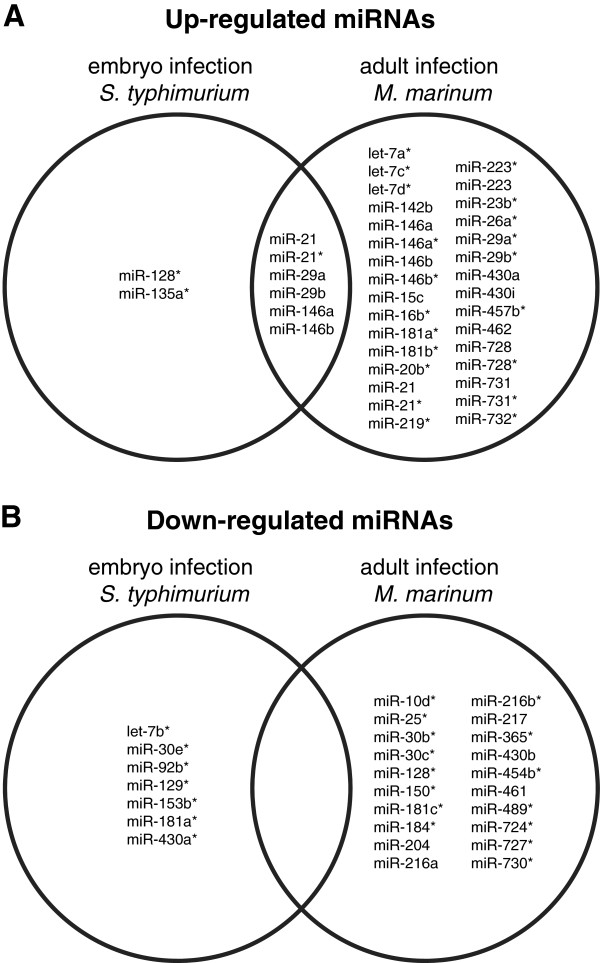
**Microarray analysis of miRNA expression during bacterial infections of zebrafish embryos and adult fish.** The Venn diagrams show the miRBase annotated miRNAs or miRNA star sequences (*) that were up-regulated **(A)** or down-regulated **(B)** during infection of zebrafish embryos with *S. typhimurium* SL1027 or infection of adult zebrafish with *M. marinum* Mma20. *S. typhimurium* infection of embryos was performed by micro-injection into the caudal vein at 28 hpf and miRNA expression was analyzed at 8 hpi in comparison with control embryos mock-injected with PBS. Adult zebrafish were infected with *M. marinum* by intraperitoneal infection and miRNA expression at 6 dpi was compared with PBS-injected controls.

In order to study the effects of bacterial infection on miRNA expression profiles in zebrafish we examined two infection conditions that are known from previous mRNA transcriptome studies to elicit a strong proinflammatory immune response: embryos at 8 hours post infection (hpi) with the *S. typhimurium* SL1027 strain and adult fish that were in the end stage of disease at 6 days post infection (dpi) with the *M. marinum* Mma20 strain [34,35]. Since embryos rely solely on innate immunity and adults also have adaptive immunity, the combination of these infection models allows determining the contribution of the two arms of the immune system to the induction of miRNAs [29,30]. To quantify microRNA gene expression profiles we used a custom designed 8×15 k Agilent zebrafish array. *S. typhimurium* infection of one-day old embryos resulted in differential expression of probes for 15 miRNAs annotated in miRBase (8 up-regulated, 7 down-regulated), while the *M. marinum*-infected adults showed differential expression of probes for 57 miRNAs (37 up-regulated, 20 down-regulated) (Figure 1A,B). The miRNA platform also contains probes for predicted hairpin structures in the zebrafish genome that might cover additional miRNAs. A large number of these probes showed differentially expression in the adult infection study (235 up-regulated, 687 down-regulated), while a much lower number was affected by infection of embryos (27 up-regulated, 37 down-regulated). Since the biological relevance of these predicted small RNAs is currently unknown, we did not consider them further in this study, but focused on the known miRNAs annotated in miRBase. Several of these miRNAs were commonly up-regulated by both of the infection conditions, including miR-21 (mature miRNA and its star sequence), miR-29a, miR-29b, miR-146a, and miR-146b (Figure 1A). Since zebrafish embryos have not yet developed adaptive immunity, it can be concluded that the context of innate immunity is sufficient to induce the expression of these miRNAs.

### miR-146a and miR-146b are induced during zebrafish infection with *S. typhimurium* and *M. marinum*

**Figure 2:**
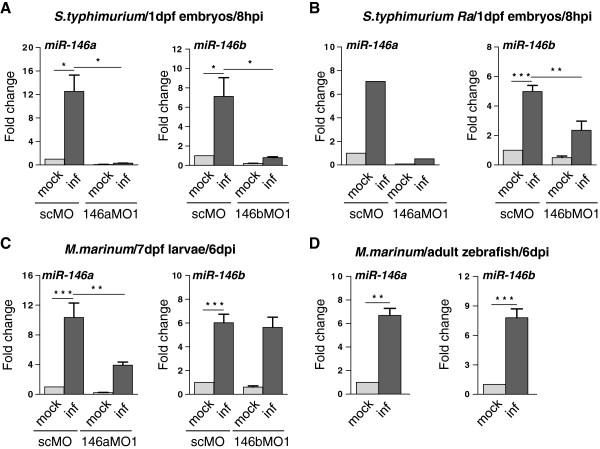
**Expression of miR-146a and miR-146b is enhanced upon bacterial infections. (A)** MiR-146a/b induction in zebrafish embryos by *S. typhimurium*. Embryos were injected with *S. typhimurium* SL1027 (inf) or PBS (mock) at 28 hpf and expression of miR-146a/b was analyzed at 8 hpi. The specificity of miRNA inductions was analyzed with morpholinos (MO) targeting miR-146a (146aMO1) or miR-146b (146bMO1). As a control a standard control morpholino (scMO) was injected. Data are the mean ± SEM of samples from two independent experiments. **(B)** MiR-146a/b induction by the attenuated *S. typhimurium* LPS mutant (Ra) strain SF1592. Experimental conditions were the same as for infection with wild type *S. typhimurium* (miR-146a: single experiment, miR-146b: mean ± SEM of three replicates). **(C)** MiR-146a/b induction in zebrafish larvae with *M. marinum* infection. Embryos were treated with miR-146a/b-targeting or control morpholinos as above, injected with *M. marinum* Mma20 (inf) or PBS (mock) at 28 hpf, and expression of miR-146a/b was analyzed in larvae at 6 dpi. Data are the mean ± SEM of samples from two independent experiments. **(D)** MiR-146a/b induction in *M. marinum*-infected zebrafish adults. Adult zebrafish were injected intraperitoneally with *M. marinum* Mma20 or mock-injected with PBS and RNA was collected at 6 dpi [35]. Data are the mean ± SEM of three fish per condition. Expression levels in all experiments were determined by TaqMan qPCR and relative expression levels are shown with the mock control set at 1. Asterisks indicate significant differences (*, P < 0.05; **, P <0.01; ***, P <0.001) tested by one-way ANOVA analysis with Tukey’s method as post-hoc test **(A**-**C)** or by an unpaired t-test **(D)**.

MiRNAs of the miR-146 family, which emerged as infection-inducible miRNAs from the microarray analysis of embryonic and adult zebrafish, have previously been linked to the innate immune response in mammalian systems [11]. To confirm the induction of these miR-146 family members we analyzed miR-146a and miR-146b expression by TaqMan qPCR analysis. MiR-222, which showed unaltered expression in the microarray study, was used as a control for normalization. In agreement with the microarray data, miR-146a and b were specifically induced in embryos at 8 hours post injection (hpi) with the *S. typhimurium* SL1027 strain (Figure 2A). Their induction was also observed in infections with an attenuated LPS mutant (Ra) strain, SF1592 (Figure 2B). Furthermore, induction of miR-146a and miR-146b was detected in zebrafish larvae at an advanced stage of *M. marinum* Mma20 infection (Figure 2C). The increase of miR-146a expression in embryos infected with *S. typhimurium* could be completely blocked with a morpholino targeting this miRNA (Figure 2A,B) and this morpholino was effective even up to the larval stage in reducing *M. marinum*-induced miR-146a expression (Figure 2C). The *S. typhimurium*-induction of miR-146b in embryos could also be reduced by morpholino treatment, (Figure 2A,B), but the miR-146b morpholino was no longer effective at the larval stage where *M. marinum* infection was analyzed. In agreement with the microarray data, induction of miR-146a and miR-146b was also confirmed in adult zebrafish infected with *M. marinum* (Figure 2D).

### Infection-inducible expression of miR-146a and miR-146b is affected by defects in signalling via the MyD88-Traf6 pathway

**Figure 3:**
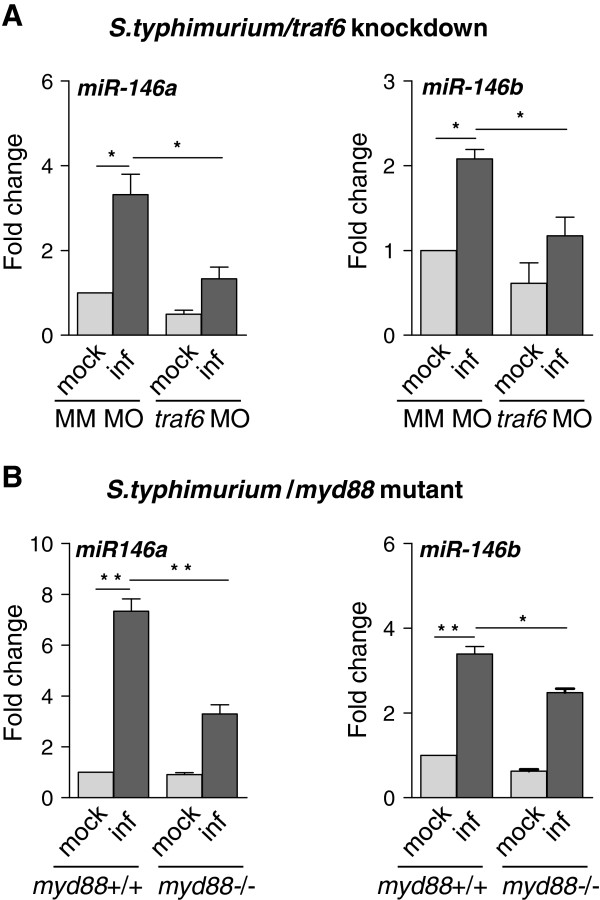
**The Traf6-MyD88 pathway is involved in up-regulation of miR-146a and miR-146b expression levels upon infection. (A)** Traf6-dependent miR-146a/b induction. Embryos were injected with *traf6* morpholino (MO) or a mismatch morpholino (MM) as a control. Embryos were infected at 28 hpf with *S. typhimurium* (inf) or mock-injected with PBS and samples were collected at 8 hpi. **(B)** MyD88-dependent miR-146a/b induction. Mutant (*myd88*-/-) and wild type siblings (*myd88*+/+) were infected with *S. typhimurium* at 28 hpf followed by sample collection at 8 hpi. Expression levels in both experiments were determined by TaqMan qPCR and relative expression levels are shown with the mock control set at 1. Data are the mean ± SEM of three replicate sample sets. Asterisks indicate significant differences (*, P < 0.05; **, P <0.01) tested by one-way ANOVA analysis with Tukey’s method as post-hoc test.

We used the *S. typhimurium* embryo infection model to investigate the dependency of miR-146a and miR-146b induction on TLR pathway genes. First, we used a previously described morpholino knockdown model for *traf6*, a central intermediate in TLR and TNF receptor signalling [36]. The *S. typhimurium*-induced expression levels of miR-146a and miR-146b were significantly lower in *traf6* knockdown embryos compared to controls (Figure 3A). Next, we analyzed miR-146a and miR-146b induction in a *myd88* mutant line [37]. Similar as under *traf6* knockdown conditions, miR-146a and miR-146b were still infection-inducible in *myd88* mutant embryos, but their expression levels were significantly higher in infected wild type siblings (Figure 3B). Therefore, we conclude that miR-146a and miR-146b induction is at least partially dependent on MyD88 and Traf6.

### MiR-146a and miR-146b knockdown does not affect leukocyte development in zebrafish embryos

**Figure 4:**
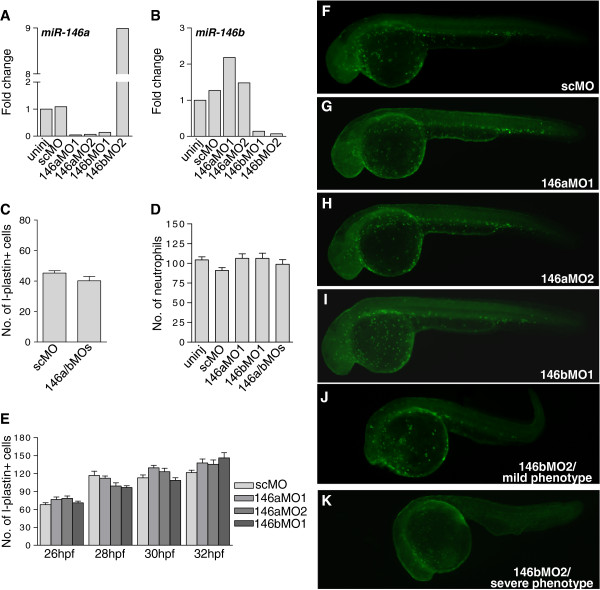
**Knockdown of miR-146a and miR-146b does not affect leukocyte development.** Embryos were injected at the 1-cell stage with morpholinos targeting miR-146a (146aMO1 and 146aMO2) or miR-146b (146bMO1 and 146bMO2) or were injected with standard control morpholino (scMO). **(A**, **B)** Confirmation of morpholino knockdown. Embryos were injected with the indicated morpholinos and RNA was collected at 2 dpf. Knockdown of miR-146a **(A)** and miR-146b **(B)** was confirmed by TaqMan qPCR. This analysis also demonstrated the specificity of morpholinos 146aMO1, 146aMO2, and 146bMO2 for their respectively target miRNAs, whereas 146bMO1 could not discriminate between the a and b forms of miR-146. 146bMO2 positively affected miR-146a expression besides blocking miR-146b expression and caused developmental aberrations **(J**, **K)**. **(C)** Quantification of L-plastin-positive leukocytes at 2 dpf. Embryos were injected with the control morpholino (scMO) or with a combination of 146aMO1 and 146bMO1 (146a/bMOs). L-plastin-labelled cells were counted manually on the left side of each embryo and the numbers present in the head, on the yolk sac, and in the tail were accumulated (n ≥ 26 embryos per group). **(D)** Quantification of Mpx-positive neutrophils at 2dpf. Embryos were injected with the indicated morpholinos. Neutrophils stained for Mpx activity were counted manually as described for L-plastin immunostaining (n ≥ 13 embryos per group). **(E)** Quantification of L-plastin-positive leukocytes at 26, 28, 30 and 32 hpf. Embryos were injected with the indicated morpholinos and L-plastin-labelled cells were counted as described above (n ≥ 16 embryos per time point). **(F-K)** Example fluorescence images of the data shown in **(E)**. The pattern of L-plastin-positive immune cells was comparable between embryos (32 hpf) injected with scMO **(F)**, 146aMO1 **(G)**, 146aMO2 **(H)**, and 146bMO1 **(I)**. 146bMO2 gave non-specific phenotypes and the number of immune cells was more variable dependent upon the severity of the phenotype **(J**, **K)**.

Loss of function studies in mice and zebrafish suggested a possible role of miR-146a in the development of myeloid cells, in addition to its proposed inhibitory effect on pro-inflammatory signalling [38]. To investigate the possible requirement of miR-146a and miR-146b for leukocyte development in zebrafish embryos, we designed two different morpholinos for each miRNA (Additional file 1: Figure S1). The efficiency of the knockdown was confirmed by TaqMan qPCR analysis, showing that basal expression of miR-146a and miR-146b (Figure 4A,B) as well as their infection inducibility (Figure 2A-C) was reduced by morpholino treatments. Both of the morpholinos designed for miR-146a (146aMO1 and 146aMO2) did not affect miR-146b expression, therefore showing specific knockdown of miR-146a only (Figure 4A,B). However, one of the miR-146b morpholinos (146bMO1) showed knockdown of both miR-146a and miR146b expression (Figure 4A,B). To assess leukocyte numbers we performed immunostaining for the pan-leukocytic marker L-plastin and histochemical staining for myeloperoxidase (Mpx) activity, a specific enzyme of neutrophils. First, we analyzed the effect of combined knockdown of miR-146a and miR-146b at 2 dpf. No differences were observed between controls and morphants in the numbers of L-plastin-stained leukocytes (Figure 4C) or Mpx-positive neutrophils (Figure 4D) at this stage. Since another study had reported an inhibitory effect of miR-146a knockdown on leukocyte development at 1 dpf [38], we next analyzed the separate effects of miR-146a and miR-146b knockdown in more detail over a critical period of leukocyte development from 26 to 32 hpf. During this period primitive myeloid cells first appear over the yolk sac, and subsequently invade the head. This first wave of primitive myeloid cells is rapidly followed by differentiation of the first erythro-myeloid precursor cells in the caudal blood island region. We performed L-plastin immunostaining with 2 h intervals over the period from 26 to 32 hpf. The total number of L-plastin-positive leukocytes showed a similar increase over this time course between control embryos and embryos injected with 146aMO1, 146aMO2, or 146bMO1 (Figure 4E-I). 146bMO2 could not be included in this quantitative analysis because it affected embryo development. Nevertheless, L-plastin positive immune cells were still present in 146bMO2 morphants with mild (Figure 4J) or severe phenotypes (Figure 4K). Based on these results, we conclude that miR-146a and miR-146b are not required for leukocyte differentiation during zebrafish embryo development.

### Combined knockdown of miR-146a and miR-146b does not have a major effect on pro-inflammatory gene expression during *S. typhimurium* infection

**Figure 5:**
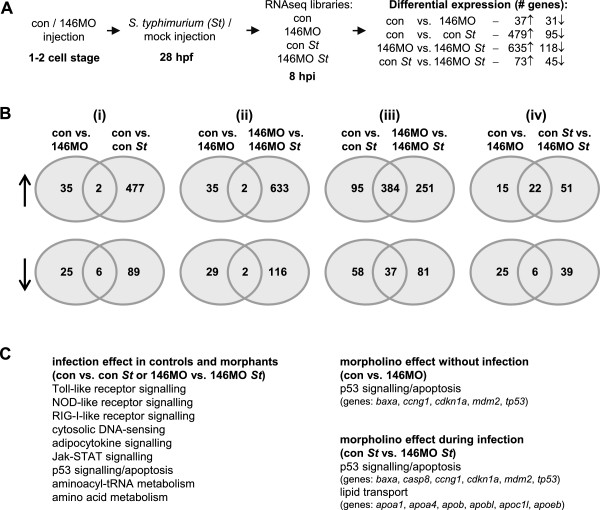
**Effect of combined miR-146a and miR-146b knockdown on the transcriptome response to *S. typhimurium *infection. (A)** Experimental set-up and DESeq analysis. Embryos treated with 146a/b morpholinos or standard control morpholino were infected with *S. typhimurium* or mock-injected with PBS, and subjected to RNAseq at 8 hpi. DESeq was used for pairwise statistical comparison of transcript count data from the four treatment groups: control/mock (con), control/infected (con *St*), 146a/bMOs/mock (146MO), and 146a/bMOs/infected (146MO *St*). The number of genes up-regulated (↑) or down-regulated (↓) is indicated. **(B)** Venn diagrams showing the overlap between differentially expressed gene sets resulting from different DESeq comparisons. Top row: up-regulated genes (↑), bottom row: down-regulated genes (↓). Venn diagrams (i) and (ii) show that there was little overlap between the morpholino effect (con vs. 146MO) and the effect of infection (con vs. con *St* or 146MO vs. 146MO *St*). Venn diagram (iii) shows strong overlap in how the control and morpholino-treated groups responded to infection. Venn diagram (iv) shows the comparison between the morpholino effect in the absence (con vs. 146MO) and in the presence (con *St* vs. 146MO *St*) of infection. **(C)** Enrichment of signalling pathways and processes revealed by gene ontology analysis. KEGG pathway analysis was performed using DAVID v6.7 (http://david.abcc.ncifcrf.gov) [42]. Pathways linked with innate immunity and metabolism were significantly enriched by infection in both the control and morpholino treatment groups (Venn diagrams i and ii above). Pathways for p53 signalling and apoptosis were enriched due to 146MO treatment both in the absence and presence of infection (overlap group in Venn diagram iv). A set of apolipoprotein genes was significantly up-regulated by infection only in the 146MO treatment group (146MO vs. 146MO *St* sector of Venn diagram iii and con *St* vs. 146MO *St* sector in Venn diagram iv).

In previous work, we observed that knockdown of a negative regulator of the immune response, the *ptpn6* phosphatase gene, resulted in a hyperinduction of pro-inflammatory gene expression during *S. typhimurium* infection [39]. Since miR-146 has also been proposed as a negative regulator of innate immunity [11], we hypothesized that miR-146 knockdown might have a similar effect. To test this hypothesis, we studied the effect of the combination of miR-146a and miR-146b morpholinos on gene expression in infected and mock-injected embryos by RNA deep sequencing (RNAseq) (Figure 5A). First we analyzed the basal expression differences between morpholino knockdown and the control in mock-injected embryos. Only 68 genes were affected by knockdown of miR-146a and miR-146b, among which 5 genes are members of the p53 signalling pathway (Figure 5B, Additional file 2: Table S1). This might reflect a non-specific effect of the miR-146 knockdown procedure, since morpholino effects on the p53 pathway are relatively common [40]. *S. typhimurium* infection had a much different effect than morpholino treatment (Figure 5B) and resulted in significant alteration of KEGG pathways related to the immune response and metabolism (Figure 5C), in agreement with previous studies [34,36,39,41]. In statistical comparison between uninfected and infected groups, the total number of infection-regulated genes was higher in miR-146 morphants (753 genes) than in control embryos (574 genes) (Figure 5A, Additional file 3: Table S2). Direct statistical comparison between the infected control and morpholino groups also showed a higher number of infection-regulated genes in miR-146 morphants (118 genes, Figure 5A). However, the only pro-inflammatory marker that was induced by infection to higher levels in miR-146 morphants compared with the controls was the *matrix metalloproteinase 9* (*mmp9*) gene (Additional file 4: Table S3). RNAseq showed a 1.5-fold higher infection-induction of this gene in miR-146 morphants, which was confirmed by qPCR analysis (Additional file 5: Figure S2). Other pro-inflammatory genes, such as *interleukin 1b* (*il1b*), CXCL and CCL family chemokines, and transcriptional regulators of the immune response were induced to similar levels in miR-146 morphants and controls. In conclusion, knockdown of miR-146a and miR-146b in zebrafish embryos did not have a strong effect on innate immunity signalling in the first 8 hours of the response to *S. typhimurium* infection, despite the increased expression of these miRNAs during this phase.

### Combined knockdown of miR-146a and miR-146b leads to increased infection-inducibility of apolipoprotein genes

**Figure 6:**
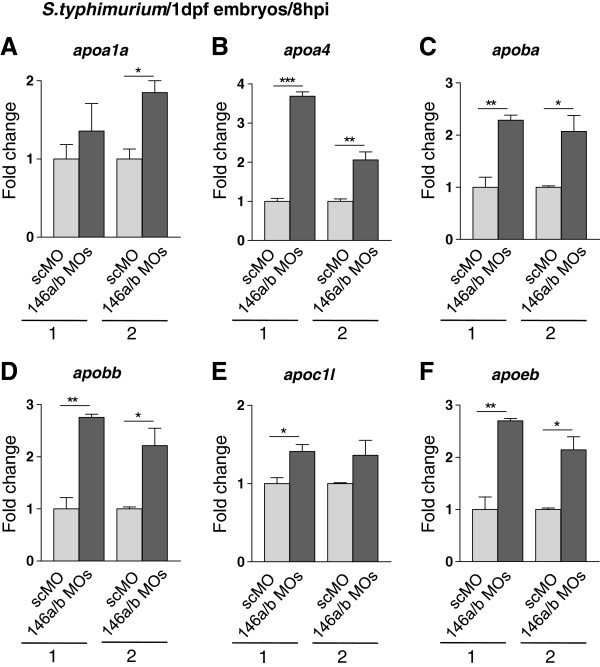
**Increased infection-induction of apolipoprotein gene expression under conditions of combined miR-146a and miR-146b knockdown. (A-F)** Embryos were injected with control morpholino (scMO) or with a combination of 146aMO1 and 146bMO1 (146a/bMOs) and infected with *S. typhimurium* as described in Figure 5. Gene expression levels of six apolipoprotein genes, *apoa1a***(A)**, *apoa4***(B)**, *apoba***(C)**, *apobb***(D)**, *apoc1l***(E)**, and *apoeb***(F)**, were analyzed by qPCR in two replicate infection experiments (1 and 2) and relative expression levels are shown with the lowest expression level set at 1. Data are the mean ± SEM of triplicate qPCR measurements. Asterisks indicate significant differences (*, P < 0.05; **, P <0.01) determined by an un-paired t-test.

Instead of a significant effect on known innate immune response genes, the RNAseq analysis revealed a possible effect on lipid transport pathways in *S. typhimurium*-infected miR-146 morphants. Six members of the apolipoprotein family (Figure 5C) were significantly induced upon *S. typhimurium* infection of miR-146 morphants but not in infected control embryos. To confirm this observation we performed qPCR analysis for the apolipoprotein gene family members (Figure 6). Only *S. typhimurium*-infected embryos were analyzed, since RNAseq analysis (Figure 5) showed an effect of miR-146 morpholinos on apolipoprotein gene expression levels in infected embryos but not in mock-injected controls. Results showed that under conditions of *S. typhimurium* infection, the expression levels of genes *apoa1a*, *apoa4*, *apoba*, *apobb*, *apoc1l*, *apoeb* were between 1.5- and 3- fold higher in miR-146 morphants compared with control embryos. Therefore, miR-146 miRNAs may be involved in fine-tuning of lipid-mediated inflammatory responses of the zebrafish embryo.

## Discussion

By microarray analysis of miRNA expression in zebrafish we found that miRNAs of the miR-21, miR-29, and miR-146 families were commonly induced by infection of embryos with *S. typhimurium* and by infection of adult fish with *M. marinum*. The induction of members of the miR-21, miR-29, and miR-146 families was in line with earlier microarray studies, which reported these along with some other miRNAs, like miR-9, miR-132, miR-147, and miR-155 as infection-inducible [13,26,43,44]. We did not detect altered expression of miR-122 and miR-194, which were found to be inducible during zebrafish infection with *Vibrio harveyi*[45]. We focused our study on the miR-146 family, which is strongly linked with immune-related diseases in human. We used zebrafish at the embryo stage, when only innate immunity is functional, as an *in vivo* model to study the role of miR-146 during bacterial infection.

The miR-146a and miR-146b sequences are conserved between zebrafish and human as well as target sites in the 3′-UTR of mRNAs of innate immune pathway genes such as *IRAK1* and *TRAF6*, which are experimentally validated targets of miR-146 [11,26]. Therefore, miR-146a and miR-146b of zebrafish may function in feedback control of TLR signalling, like the human and murine counterparts [11,26]. To study the pathway by which miR-146 expression is induced in zebrafish embryos upon infection we used embryos in which TLR signalling was disrupted by morpholino knockdown of *traf6* or by mutation of *myd88*. The induction levels of miR-146a and miR-146b upon *S. typhimurium* infection were reduced under conditions of *traf6* or *myd88* deficiency, but induction was not completely abolished. In the case of *traf6* deficiency, the residual induction of miRNA expression may be due to a partial morpholino knockdown effect. We have previously shown that mutation of *myd88* strongly affects the innate immune response to *S. typhimurium* infection, but that innate immune genes can still be induced to lower levels in the absence of functional MyD88 [37]. Therefore, it is likely that both the MyD88-Traf6-dependent pathway and parallel MyD88-independent signalling routes contribute to the infection-induced expression of miR-146.

A recent study by Ghani et al. [38] suggested miR-146a to be required for myeloid cell differentiation in mouse and zebrafish. They reported that miR-146a morpholino knockdown caused an almost complete absence of myeloid cells in zebrafish embryos at 1 dpf. However, in our analysis we found no evidence for an inhibitory effect of miR-146 deficiency on myeloid cell development. We used two morpholinos for miR-146a (one of which was the same as a miR-146a morpholino used by Ghani et al. [38]), and verified the knockdown effect by TaqMan qPCR, which showed that the morpholinos effectively inhibited both the basal expression and the infection-induced expression. By immunostaining for the pan-leukocytic marker L-plastin we detected no differences in myeloid cell development between miR-146a morphants and controls over an elaborate time course between 26 and 32 hpf, which comprises the critical embryonic stages when myeloid cells differentiate and enter the circulation. Furthermore, no effect on neutrophil differentiation at 2 dpf was detected. For miR-146b we also did not observe an effect on leukocyte development with two different morpholinos targeting this miRNA. Ghani et al. [38] used mRNA in situ hybridization for detection of L-plastin, which might explain the difference with our study in which we used a sensitive immunolabelling method, which is widely used for detection and quantification of myeloid cells in zebrafish embryos [46,47]. Our conclusion that myeloid development in zebrafish embryos is not inhibited by miR-146a or miR-146b deficiencies is in line with the phenotype of miR-146a knockout mice, which also were not impaired in myeloid differentiation but in fact showed hyperproliferation of myeloid cells leading to autoimmunity [26].

Knockout mice of miR-146a are hyperresponsive to LPS, showing increased up-regulation of pro-inflammatory cytokines, such as TNF and IL-6 [26]. We used *S. typhimurium* infection of zebrafish embryos, which results in strong pro-inflammatory gene induction, to analyze the knockdown effect of miR-146a and miR146b by RNAseq analysis. We used a combination of morpholinos against miR-146a and miR-146b in the RNAseq study to avoid that the two miRNAs might compensate for each other’s loss-of-function, as their mature sequences differ only by two nucleotides. Several genes in the p53 pathway, including p53 itself, were up-regulated in miR-146 morphants as compared to controls under infected as well as non-infected conditions. This might be attributed to the well known off target effects of morpholino oligonucleotides [40]. However, as miR-146 has been frequently linked with cancer, a direct effect on the p53 pathway cannot be excluded [18,48,49]. In fact, one of the p53 pathway genes up-regulated by miR-146 knockdown, *cdkn1a* (p21), is an experimentally validated target of miR-146a in human [50]. In total we found 73 genes which were significantly up-regulated in miR-146 infection as compared to control infection. Besides *cdkn1a*, only one other gene, *fibrinogen beta chain* (*fgb*), showed an overlap with the predicted targets of zebrafish miR-146a and miR-146b in miRBase. Fibrinogen has roles in cell adhesion, hematopoiesis, and in coagulation and complement cascades associated with primary defence against bacterial infections [51]. Expression levels of other known targets of miR-146 involved in innate immunity, such as *irak1*, *traf6, irf5* and *stat1*, were not affected, but this was not unexpected since miRNAs can act by translational inhibition.

The matrix metalloproteinase gene *mmp9* is one of the most strongly induced pro-inflammatory markers in *S. typhimurium* infection [34]. The combined morpholino knockdown of miR-146a and miR-146b led to slightly increased induction of this gene during *S. typhimurium* infection; however, this induction of *mmp9* was not accompanied by a general hyperinduction of other pro-inflammatory markers in the RNAseq analysis. *MMP9* is not a predicted target gene of miR-146 in human or zebrafish, but human *MMP9* was found to be down-regulated upon miR-146a/b overexpression in MDA-MB-231 breast cancer cells and in THP-1 macrophages [20,52]. This down-regulation was suggested to occur via TLR-mediated and NFκB-dependent pathways rather than by direct targeting of *MMP9*[20,52]. Likewise, the induction of zebrafish *mmp9* under miR-146a/b knockdown conditions might be an indirect consequence of effects on upstream signalling proteins. In agreement, we have previously shown that *mmp9* induction by *S. typhimurium* infection is mediated by Traf6, which is a known target of miR-146 [11,26,36].

By targeting components of TLR signalling, miR-146 has been shown to function as a negative regulator of the innate immune response in mammals [11,26]. However, in our study of *S. typhimurium* infection in zebrafish embryos, miR-146 knockdown did not have a strong impact on the induction of proinflammatory genes. Notably, the effect of miR-146 knockdown was minor in comparison with knockdown analysis of *ptpn6*, which encodes a SH2-domain phosphatase that functions as a negative regulator of innate immunity [53,54]. In the same experimental set-up, *S. typhimurium* infection of zebrafish embryos after knockdown of *ptpn6* resulted in hyperinduction of *mmp9* and a wide range of cytokines, other immune effectors genes, and transcriptional regulators of the immune response [39], while in the case of miR-146 knockdown only *mmp9* was slightly higher induced. Furthermore, hyperinflammation in *ptpn6* morphants impaired control of *S. typhimurium* infection [39], while miR-146 knockdown had no such effect (data not shown). These results support that in the early response of zebrafish embryos to *S*. *typhimurium* infection Ptpn6 functions as a much stronger negative feedback regulator than miR-146. This would be consistent with the idea that miRNAs function in a subtle fine-tuning of the immune response [1].

While the overall knockdown effect observed in our RNAseq analysis was relatively minor, apolipoprotein-mediated lipid transport emerged as an infection-inducible pathway under miR-146 knockdown conditions. Numerous studies have linked apolipoproteins to immunoregulation and host defence [55-57]. MiR-146a has been suggested to be involved in negative regulation of oxidized low-density lipoprotein-(LDL) accumulation in macrophages [52]. Lipid accumulation in macrophages is associated with the inflammatory processes that lead to atherosclerosis. The expression of miR-146a was found to be down-regulated when THP-1 macrophages were stimulated with oxidized LDL. Furthermore, miR-146 overexpression reduced intracellular LDL cholesterol content and secretion of IL6, IL8, and MMP9 via TLR4-mediated signalling. A similar effect on LDL accumulation was observed by silencing miR-155, another important miRNA regulator of immune processes [58]. Based on its regulatory role in lipid accumulation miR-146a has been proposed as a potential therapeutic candidate for atherosclerosis treatment [52]. Our results support the inhibitory function of miR-146 in lipid-mediated inflammatory responses and the proposed application as a therapeutic target.

## Conclusion

Recent studies have demonstrated the involvement of miRNAs in immune processes and their link to inflammatory disorders and have increased interest to find the molecular pathways responsible for miRNA action [1]. MiR-146 has been recognized as a modulator of the innate and adaptive immune responses in mammals [11,26,28]. In a microarray analysis of miRNA expression in zebrafish, both of the miR-146 family members, miR-146a and miR-146b, were found to be inducible by *S. typhimurium* and *M. marinum* infections. The miR-146 family members were commonly induced during infections of embryos and adult fish, along with miRNAs of the miR-21 and miR-29 families, which also have been implicated in immunity and infection. The induction of these miRNAs in embryo infection models links them specifically with the innate immune response, as adaptive immunity is not yet functional at early developmental stages. We exploited the embryo model as an *in vivo* system to investigate the role of miR-146 in the innate immune response to *S. typhimurium* infection. Induction of miR-146a and miR-146b by infection was shown to be affected by deficiencies in Traf6 and Myd88, which are central intermediates of Toll-like receptor and cytokine signalling pathways. MiR-146 has previously been implicated in negative feed-back regulation of these pathways [11,26], similar to a number of signalling proteins, including the protein-tyrosine phosphatase Ptpn6 [53,54]. However, whereas knockdown of the *ptpn6* gene caused hyperinflammation in zebrafish embryos [39], knockdown of miR-146 in *the S. typhimurium* embryo infection model had no major effect on pro-inflammatory gene expression or on the expression of transcriptional regulators and signal transduction components of known immune response mediators. In contrast, several members of the apolipoprotein gene family (*apoa1a*, *apoa4*, *apoba*, *apobb*, *apoc1l*, and *apoeb*) were infection inducible only under miR-146 knockdown conditions. The apolipoprotein family has been linked to immunoregulation and host defence in many studies [55,56]. Consistent with a previous report proposing miR-146a as a negative regulator of LDL accumulation in human macrophages and a therapeutic target for atherosclerosis treatment [52], our results suggest a possible function of miR-146 in regulating apolipoprotein-mediated lipid transport under the inflammatory conditions of *S. typhimurium* infection.

## Methods

### Zebrafish husbandry

Zebrafish were handled in compliance with the local animal welfare regulations and maintained according to standard protocols (zfin.org). The breeding of adult fish was approved by the local animal welfare committee (DEC) of Leiden University. All protocols adhered to the international guidelines specified by the EU Animal Protection Directive 2010/63/EU. Embryos from the zebrafish AB/TL line were used for the infection experiments. In addition, an infection experiment was performed using embryos from a *myd88* mutant line and wild type siblings as a control [37]. Embryos were grown at 28–30°C in egg water (60 μg/ml Instant Ocean sea salts). For the duration of bacterial injections embryos were kept under anesthesia in egg water containing 200 μg/mL tricaine (Sigma-Aldrich). Significance cut-offs for DEseq analysis were set at: absolute fold change ≥1.5 and adjusted P-value ≤ 0.1. Embryos used for immunostaining and Myeloperoxidase (Mpx) assay were kept in egg water containing 0.003% 1-phenyl-2-thiourea (Sigma-Aldrich) to prevent melanization.

### Infection experiments

*S. typhimurium* infections were performed using strain SL1027 and its isogenic LPS Ra mutant derivative SF1592, carrying the DsRed expression vector, pGMDs3 [59]. *M. marinum* infections were performed with strain Mma20 expressing mCherry in pSMT3 vector [35,60]. Bacteria were grown and prepared for injections as previously described and microinjected into the caudal vein of embryos at 28 hours post fertilization (hpf), using a dose of 200–250 CFUs of *S. typhimurium* or 100 CFU of *M. marinum* per embryo [61]. As a control, embryos were mock-injected with PBS. After injections, embryos were transferred to fresh egg water and incubated at 28°C. Adult zebrafish infected with the *M. marinum* Mma20 strain (6 dpi) and PBS-injected control fish were from a previous study [35].

### RNA/miRNA isolation and quality check

Embryos were snap frozen in liquid nitrogen and kept at -80°C. Adult zebrafish were homogenized in liquid nitrogen and kept in portions of 50–100 μg of powdered tissue at -80°C. Total RNA was isolated from frozen embryos or tissue homogenates using the miRNeasy Mini kit (Qiagen) with an on-column DNA purification with RNase Free DNase set (Qiagen). RNA quality of samples for RNAseq analysis was checked with an Agilent Bioanalyzer 2100 using the RNA 6000 Nano series Kit (Agilent, Santa Clara, CA, USA). All samples had a RNA integrity value (RIN) of 10.

### Microarray analysis of miRNAs

Custom-designed 8×15 k microarray slides were ordered from Agilent Technologies. The 15 k custom design was obtained from Edwin Cuppen and Eugene Berezikov (Hubrecht Institute, Utrecht, The Netherlands) and has been submitted into the Gene Expression Omnibus (GEO) database (GPL 15403). The 15 k design contained a duplicate of 7604 probes of 60-oligonucleotide length. The probes consisted of 2x22 nucleotide sequences antisense to mature miRNAs separated by a spacer of 8 nucleotides (CGATCTTT) and with a second spacer with the same sequence at the end. From 7604 probes 546 were designed for left (5′) and right (3′) arms of the hairpins of zebrafish miRNAs that are known in miRBase, while the remainder 7058 probes corresponded to predicted hairpin structures in the zebrafish genome that might include additional miRNAs. Total RNA, including microRNA, was extracted from pools of 20–30 embryos or from individual adult fish using the miRNeasy Mini Kit® (Qiagen). Three biological replicates were used for each condition. RNA labelling was carried out with the miRCURY™ LNA microRNA, Hy3™/Hy5™ Power Labelling kit (Exiqon) using 1 μg of total RNA according to the manufacturer’s instructions. RNA samples from infected embryos or adults were labelled with Hy3 and hybridized against Hy5-labelled RNA samples from PBS-injected controls. The dual color hybridization of the microarray chips was performed according to Agilent protocol GE2_105_Jan09 (http://www.Agilent.com) for two-color microarray-based gene expression analysis except that hybridization and washing was performed at 37°C. The arrays were scanned with DNA Microarray Scanner G2505B from Agilent Technologies. The arrays were scanned twice with 10% PMT and 100% PMT laser power. Microarray data was processed from raw data image files with Feature Extraction Software 9.5.3.1 (Agilent Technologies). The XDR function was used to extend the dynamic range. Processed data were subsequently imported into Rosetta Resolver 7.1 (Rosetta Biosoftware, Seattle, Washington) and subjected to default ratio error modelling. Ratio results from control vs. infected replicates were combined using the default ratio experiment builder. Significance cut-offs for the ratios of infected versus control were set at 1.5-fold change at P ≤ 10 - 4. Significance cut-offs for DEseq analysis were set at: absolute fold change ≥1.5 and adjusted P-value ≤ 0.1. The raw data have been submitted to GEO under accession number GSE45410.

### Morpholino knockdown

Morpholino oligonucleotides (GeneTools) were diluted to the desired concentration in 1× Danieau buffer (58 mM NaCl, 0.7 mM KCl, 0.4 mM MgSO_4_, 0.6 mM Ca(NO_3_)_2_, 5.0 mM HEPES; pH 7.6) containing 1% phenol red (Sigma-Aldrich) and approximately 1 nl was injected at the 1–2 cell stage using a Femtojet injector (Eppendorf). For knockdown of miR-146a and miR-146b two morpholinos were used against each of them. The first morpholino for miR-146a (146aMO1: 5′ACCATCTATGGAATTCAGTTCTCAG3′) targets the miRNA guide strand and the second morpholino (146aMO2: 5′GAGCCCAUAGAUGAACUUUUCAUGA3′) overlaps with the star strand and the dicer cleavage site on the star strand (Additional file 1: Figure S1A). For miR-146b, the first morpholino (146bMO1: 5′GACACCCTTGGAATTCAGTTCTCAA3′) also targets the guide strand, and the second morpholino (146bMO2: 5′CGTGGGCTGAATATAAAGCAGACAC3′) overlaps with both dicer cleavage sites and part of the star strand (Additional file 1: Figure S1B). All miR-146 morpholinos could be used at a concentration of 0.75 mM without causing morphological defects, except146b-MO2, which was highly toxic. Another morpholino design for miR-146b was not recommended by GeneTools. For *traf6* knockdown we used a previously described morpholino [36]. As a control the standard control morpholino (scMO) from GeneTools was used as previously described [39].

### Detection of leukocytes

Embryos were fixed in 4% paraformaldehyde (PFA) in PBS. Immunofluorescence detection of leukocytes was performed with a 1:500 dilution of polyclonal rabbit Ab against L-plastin [57] and Alexa Fluor 488 goat anti-rabbit IgG secondary Ab (Molecular Probes), as previously described [62]. Fluorescence images were taken with a Leica MZ16FA stereo fluorescence microscope equipped with a DFC420C digital color camera. Histochemical detection of neutrophils was performed by Mpx activity staining using the Peroxidase Leukocyte Kit (Sigma-Aldrich) as previously described [62].

### RNAseq analysis

For RNAseq analysis, embryos were injected with a combination of 146aMO1 and 146bMO1, or with the scMO. Subsequently, at 28 hpf they were infected with *S. typhimurium* or mock-injected with PBS, and RNA was isolated from pools of at least 50 embryos at 8 hours post injection (hpi). Two independent experiments were performed for RNAseq analysis of biological duplicates. A total of 3 μg of RNA was used to make RNAseq libraries using the Illumina TruSeq RNA Sample Preparation Kit v2 (Illumina Inc., San Diego, USA). In the manufacturer’s instructions two modifications were made. In the adapter ligation step 1 μl instead of 2.5 μl adapter was used. In the library size selection step the library fragments were isolated with a double Ampure XP purification with a 0.7× beads to library ratio. The resulting mRNA-Seq library was sequenced using an Illumina HiSeq2000 instrument according to the manufacturer’s description with a read length of 2 × 50 nucleotides. Image analysis and base calling was done by the Illumina HCS version 1.15.1. Sequence reads were quality trimmed using the quality_trim module in the CLCbio Assembly Cell v4.0.6. Filtered reads were mapped to Ensembl transcripts (Zv9_63) using the ref_assemble_short module in the CLCbio Assembly Cell v4.0.6. Accumulation of transcripts to Ensembl genes was done by first converting the mapping files to a table with the assembly_table module in the CLCbio Assembly Cell v4.0.6. Secondly a custom script was used that sums all reads belonging to a transcript. Non-uniquely mapped reads were divided between transcripts according to their ratio of uniquely mapped reads. Finally, read counts of transcripts belonging to the same gene were summed to obtain count data at Ensembl gene level. Fold-change and differential expression significance values were calculated from gene level read counts using the DESeq package (version 1.8.3) available in Bioconductor (version 2.10). DESeq utilizes a negative binomial distribution for modeling read counts per gene and implements a method for normalizing the counts [63]. Significance cut-offs for DEseq analysis were set at: absolute fold change ≥1.5 and adjusted P-value ≤ 0.1. The raw data have been submitted to GEO under accession number GSE45410.

### Quantitative RT-PCR analysis

For quantification of miR-146a/b expression levels, RT-PCR reactions were performed using a TaqMan microRNA reverse transcription kit (Applied Biosystem) according to the manufacturer’s instructions. Briefly, 10 ng of total RNA was reverse transcribed using 5× RT primers (Custom TaqMan small RNA Assay-Applied Biosystem) in a total reaction volume of 15 μl. Reactions were kept on ice for 5 min and then were transferred to the thermal cycler for incubations at 16°C and 42°C for 30 min each, followed by an incubation at 85°C for 5 min. Quantitative RT-PCR with 0.665 μl cDNA input per reaction was performed using a Custom TaqMan small RNA Assay for each miRNA and a TaqMan Universal PCR Master Mix (Applied Biosystem) in a total of 10 μl per reaction. Cycle threshold values were calculated under the parameters of 40 cycles of 10 min at 95°C, 15 sec at 95°C and 60 sec at 60°C. All reactions were performed in triplicate. For normalization, miR-222, which showed no changes in response to bacterial challenge, was taken as reference. Results were analyzed using the ∆∆Ct method. Quantification of *mmp9* and apolipoprotein gene expression was performed as previously described [39], using the *ppial* gene for normalization and the primers in Additional file 6: Table S4.

### Availability of supporting data

The data sets supporting the results of this article are available in the Gene Expression Omnibus repository under accession number GSE45410 (http://www.ncbi.nlm.nih.gov/geo/query/acc.cgi?acc=GSE45410).

## Competing interests

The authors declare that they have no competing interests.

## Authors’ contributions

AO and ZK performed experiments, analyzed data, prepared figures and wrote the manuscript. VL performed experiments and analyzed data. JJYR analyzed data. MM and HPS provided materials and resources for this study and gave input for the manuscript. AHM conceived and supervised the study and wrote the manuscript. All authors read and approved the final version.

## Supplementary Material

Additional file 1: Figure S1. Target sites of miR-146a and miR-146b morpholinos on their respective miRNAs. The stem-loop sequences of the zebrafish miR-146a and miR-146b homologs, dre-miR-146a (A) and dre-miR-146b (B) are shown with the miRNA guide strand in lower case. The activity of miRNAs can be blocked using morpholinos complementary to the miRNA guide strand or to the Drosha or Dicer nucleolytic processing sites of the primary miRNA or pre-miRNA (http://www.gene-tools.com). Regions targeted by the morpholinos are indicated in blue (146aMO1 and 146bMO1) or yellow (146aMO2 and 146bMO2), and overlap between two morpholino regions is shown in green. Click here for file

Additional file 2: Table S1. Effect of miR-146a and miR-146b knockdown on gene expression in the absence of infection. The table lists the genes showing significantly up- or down-regulated expression under knockdown conditions of miR-146a and miR-146b in comparison with control embryos (experimental set-up shown in Figure 5A). Significance cut-offs were: -1.5 ≤ fold change ≤ 1.5 and adjusted P-value ≤ 0.1 in statistical analysis with DESeq. Some genes were undetectable in one of the samples, resulting in an infinite fold change, which is indicated in the table as Inf or Inf down for up- and down-regulated genes, respectively. Genes are annotated by Ensembl ID, description and gene symbol. Click here for file

Additional file 3: Table S2. Effect of *S. typhimurium* infection on gene expression in control embryos and under conditions of miR-146a and miR-146b knockdown. The table lists the genes showing significantly up- or down-regulated expression due to *S. typhimurium* infection in embryos injected with a standard control morpholino or with a combination of morpholinos targeting miR-146a and miR-146b (experimental set-up shown in Figure 5A). Significance cut-offs were: -1.5 ≤ fold change ≤ 1.5 and adjusted P-value ≤ 0.1 in statistical analysis with DESeq. Significant values are marked yellow. Some genes were undetectable in one of the samples, resulting in an infinite fold change, which is indicated in the table as Inf or Inf down for up- and down-regulated genes respectively. Genes are annotated by Ensembl ID, description and gene symbol. Click here for file

Additional file 4: Table S3. Effect of miR-146a and miR-146b knockdown on gene expression during *S. typhimurium* infection. The table lists the genes showing significantly up- or down-regulated expression in *S. typhimurium*-infected embryos under knockdown conditions of miR-146a and miR-146b in comparison with *S. typhimurium*-infected control embryos (experimental set-up shown in Figure 5A). Significance cut-offs were: -1.5 ≤ fold change ≤ 1.5 and adjusted P-value ≤ 0.1 in statistical analysis with DESeq. Some genes were undetectable in one of the samples, resulting in an infinite fold change, which is indicated in the table as Inf or Inf down for up- and down-regulated genes, respectively. Genes are annotated by Ensembl ID, description and gene symbol. Click here for file

Additional file 5: Figure S2. Increased *mmp9* expression in *S. typhimurium* infection under knockdown conditions of miR-146a and miR-146b. Embryos were injected with control morpholino (scMO) or with a combination of 146aMO1 and 146bMO1 (146a/bMOs) and infected with *S. typhimurium* or mock-injected with PBS as described in Figure 5. Gene expression of *mmp9* was analyzed by qPCR and relative expression levels are shown with the mock control set at 1. The *mmp9* induction level after *S. typhimurium* infection was significantly higher in miR-146a/miR-146b morphants than in control embryos, consistent with the results of RNAseq analysis (Additional file 4: Table S3). Data are the mean ± SEM of samples from two independent experiments. Asterisks indicate significant differences (*, P < 0.05; **, P <0.01) tested by one-way ANOVA analysis with Tukey’s method as post-hoc test. Click here for file

Additional file 6: Table S4. Primer sequences for qPCR analysis of apolipoprotein genes. Forward and reverse primer sequences for apolipoprotein genes A-1a, A-IV, Ba, Bb, C1-like, and Eb. Click here for file
