# Supplementary figures and images for: MicroRNA-146 function in the innate immune transcriptome response of zebrafish embryos to Salmonella typhimurium infection
Source: BMC Genomics. 2013 Oct 10;14:696. doi: 10.1186/1471-2164-14-696 (PMC3852110; doi:10.1186/1471-2164-14-696)

## A stem-loop dre-miR-146a

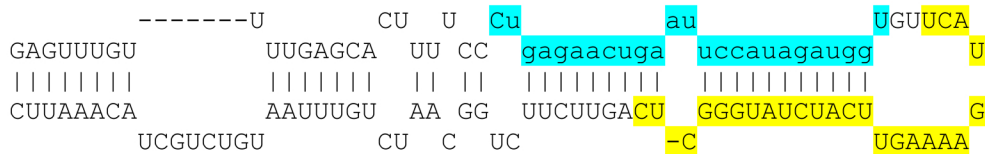

## B stem-loop dre-miR-146b

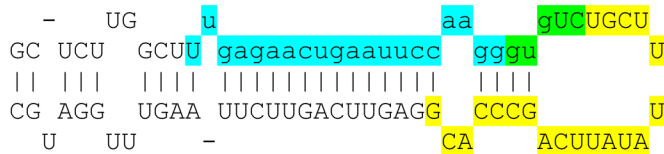

Supplement: Additional file 1: Figure S1. — Target sites of miR-146a and miR-146b morpholinos on their respective miRNAs. The stem-loop sequences of the zebrafish miR-146a and miR-146b homologs, dre-miR-146a (A) and dre-miR-146b (B) are shown with the miRNA guide strand in lower case. The activity of miRNAs can be blocked using morpholinos complementary to the miRNA guide strand or to the Drosha or Dicer nucleolytic processing sites of the primary miRNA or pre-miRNA (http://www.gene-tools.com). Regions targeted by the morpholinos are indicated in blue (146aMO1 and 146bMO1) or yellow (146aMO2 and 146bMO2), and overlap between two morpholino regions is shown in green. [file 1471-2164-14-696-S1.pdf]

Fold change

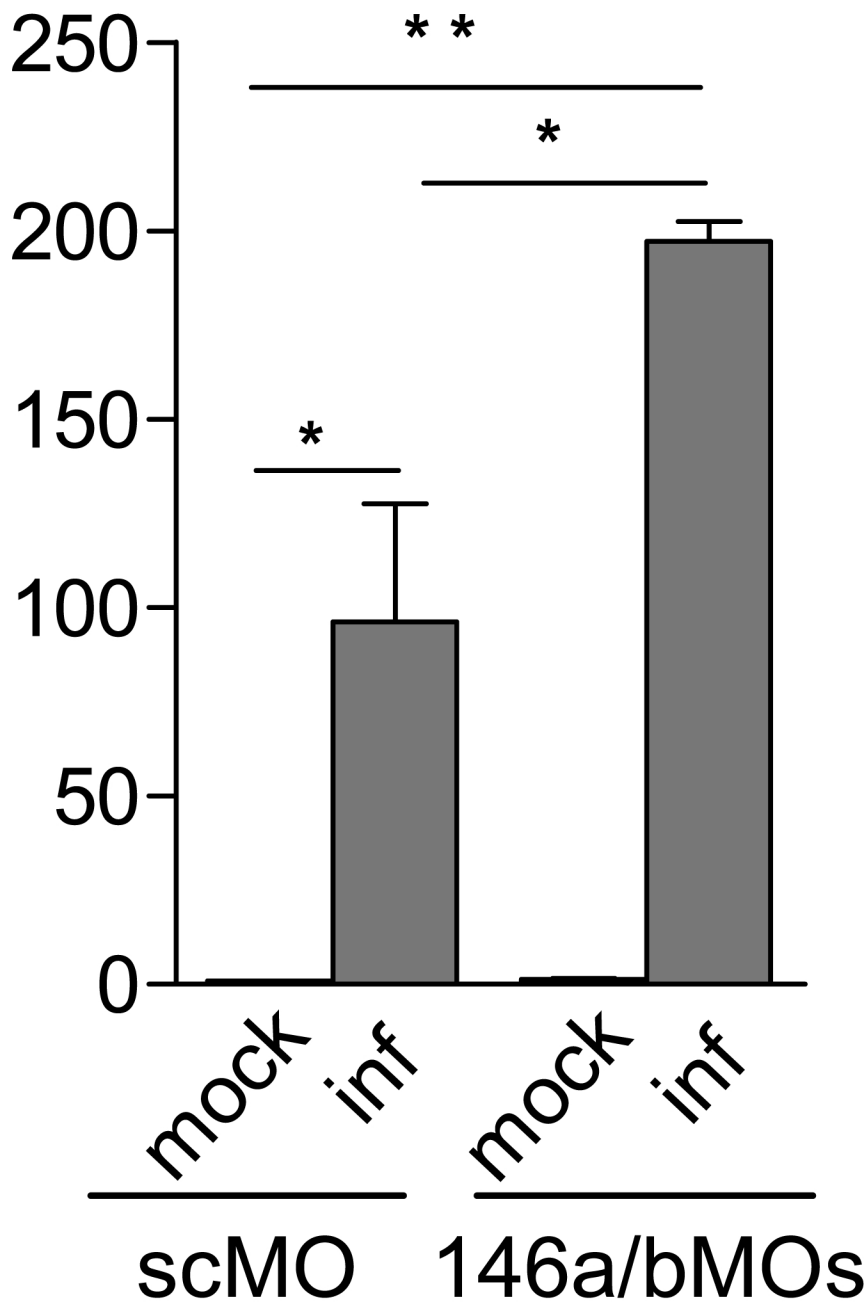

Supplement: Additional file 5: Figure S2. — Increased mmp9 expression in S. typhimurium infection under knockdown conditions of miR-146a and miR-146b. Embryos were injected with control morpholino (scMO) or with a combination of 146aMO1 and 146bMO1 (146a/bMOs) and infected with S. typhimurium or mock-injected with PBS as described in Figure 5. Gene expression of mmp9 was analyzed by qPCR and relative expression levels are shown with the mock control set at 1. The mmp9 induction level after S. typhimurium infection was significantly higher in miR-146a/miR-146b morphants than in control embryos, consistent with the results of RNAseq analysis (Additional file 4: Table S3). Data are the mean ± SEM of samples from two independent experiments. Asterisks indicate significant differences (*, P < 0.05; **, P <0.01) tested by one-way ANOVA analysis with Tukey’s method as post-hoc test. [file 1471-2164-14-696-S5.pdf]
